# Target2 imbalances and poverty in the eurozone

**DOI:** 10.1007/s00191-022-00797-0

**Published:** 2022-11-12

**Authors:** Rosaria Rita Canale, G. Liotti

**Affiliations:** 1 Department of Business and Economics, University of Naples ‘Parthenope’, Naples, Italy; 2 Department of Economics, University of Messina, Messina, Italy

**Keywords:** Poverty, Target2, Interest rates, Growth, Eurozone, Dynamic panel data, F45, I30, C33

## Abstract

This paper aims to investigate the relationship between external imbalances and poverty in the Eurozone. The former are registered through the Target2 (T2) settlement mechanism and can be assimilated into changes in official reserves to cover the balance of payments disequilibrium in a fixed exchange rate regime. The presence of T2 discrepancies has led to differences in interest rates and increased distances in general living conditions inside the Eurozone. An empirical investigation implemented in 11 Eurozone countries reveals that T2 is negatively correlated with poverty, therefore allowing for an interpretation that approximates balance of payment crisis models. Results that appear to be robust to several control variables suggest that the policy framework of the Eurozone—in the absence of a compensatory mechanism—should be revised towards centralised fiscal instruments and anti-speculative monetary interventions.

## Introduction

The Eurozone is a currency area that has always experienced alternating fortunes. From its creation until 2008, a generalised process of convergence seemed to occur (Blanchard and Giavazzi 2002). With the eruption of the financial crisis and its subsequent transformation into a sovereign bond crisis, a number of imbalances emerged that revealed the fragility of the currency union (Calmfors et al. 2012).

Among these imbalances, those arising from the balance of payments play a special role. They are favoured by a policy structure in which monetary policy is centralised and fiscal policy is left to the management of individual states (Bini Smaghi 2011). It threatens to be a source of persistent and self-fulfilling disequilibria (De Grauwe and Ji 2013).

This paper investigates how these external imbalances affect divergences inside 11 Eurozone countries, connecting the Target2 (T2) balance of payments mechanism of compensation with poverty. Target2 can be assimilated into the change in official reserves necessary to cover the balance of payments disequilibrium in a fixed exchange rate regime. They arise as a symptom of expected divergences and are worsened by investors’ speculative behaviour. Its effect on interest rates seems to mirror those that occur during a currency crisis in a fixed exchange rate regime. However, difficulties do not arise in the way that first-generation models predicted (Krugman 1979) because of the exhaustion of foreign reserves − T2 imbalances can potentially reach infinite levels − but rather because of the automatic effects on interest rates of net capital flows. This result resembles those presented in the second (Obstfeld 1986a and 1986b) and third (Eichengreen and Hausmann 1999) generation currency crisis models in which national central banks raise interest rates in an attempt to preserve exchange rate parity and hinder speculative behaviour by investors. When the loss in terms of internal equilibrium is too high, the exchange rate becomes free to fluctuate. However, in the Eurozone case, the eventual abandonment of the fixed exchange regime does not occur but rather continues to feed distances across countries.

We measured such distance through poverty. To measure poverty, Eurostat refers to two main categories of indicators: the first one captures relative poverty and the second captures absolute poverty. That measuring relative poverty is “monetary poverty” or “people at risk of poverty after social transfers” and represents the share of the total population with an equivalised disposable income below the at-risk-of-poverty threshold of 60% of the national median-equivalised disposable income after social transfers. Absolute poverty is measured by “material deprivation,” or the percentage of the population prevented by a lack of resources from having a “decent” life (see below for a detailed explanation; Eurostat 2016). Both indicators consider the “society” for which they are calculated since they refer to geographically and historically defined assets. However, while “monetary poverty” is country-specific and can be considered a measure of inequality as the number of poor people is calculated in respect to a threshold, “material deprivation” has the advantage of being comparable across nations that belong to the same economic area (Kenworthy 2011; Darvas et al. 2014; Crettaz 2015). Unlike inequality, which does not necessarily register conditions of difficulty for individuals, material deprivation measures the dimension of impoverishment in a society, which has to be taken into consideration when defining an economy as “advanced.” When connected with external imbalances, it allows measuring the effect of net capital flows inside the Eurozone on poverty in each single country useful to reflect on the policy structure of the currency area. It is an irrevocable fixed exchange rate regime in which the centralised monetary policy, together with fiscal constraints, seem to support disparities rather than convergence. The chosen point of view reconciles the two interpretations relating to T2 imbalances. They are the manifestation of a balance of payments crisis (Cesaratto 2017), which persists − despite the introduction of refinancing operations (RO) to support the banking sector − because of the restrictions required to respect fiscal parameters and the unwillingness of the European Central Bank (ECB) to assume the role of the unlimited purchaser of public debt (Febrero et al. 2018).

Our investigation follows two routes. First, we ascertain how the relationship between T2 imbalances and poverty arises. T2 balances lead to differences in interest rates, whatever the policy rate and the non-conventional instruments the ECB implements (Rochon and Setterfield 2007; Jorda et al. 2020). These differences create gaps in growth and therefore in absolute poverty in each country. Second, we conduct an empirical investigation through a dynamic panel technique, the pooled mean group (PMG) estimator, in 11 Eurozone countries from 2008 to 2019. The starting date follows the occurrence of external imbalances that, up to that year had been equal to zero. We use the year average of T2 because of the availability of data about poverty. Despite the limited number of observations, it provides an insight into the proposed relationship and – differently from vector error correction (VECM) models – release consistent results in presence of heterogeneity across panel members. However, results appear to be robust even when introducing—one by one—a number of control variables affecting absolute poverty, capturing the institutional conditions of each single country as well as Eurozone policy choices.

We follow, therefore, rather than the first generation currency crisis models predicting that deteriorating fundamentals *push* the economy into the currency crisis, the second and third generation models, according to which expectations of deteriorating fundamentals are *ex-post* factors to *pull* the economy into a crisis. If the commitment to preserve the parity if very high – as in the case of the EMU- the cost in term of output volatility is very high as well (Obstfeld 1986a, 1986b, Saxena 2004, italics borrowed from Flood and Marion 2000). Our main argument pivots on the different effects on interest rates arising from T2 imbalances. We are not in search of the whole set of determinants of target imbalances, but rather of a correlation between T2 and interest rates, following the idea that the higher the commitment toward the fixed exchange rate the higher the cost in terms of internal equilibrium. In fact, the increase in interest rates, have opposite sign effect on poverty through its effect on output and employment and therefore on the number of those at the lower end of income distribution. Indeed, the indicator of absolute poverty can be considered a proxy of the per capita income growth rate of the poorest living in the countries considered. They do not have access to international capital markets and are therefore deprived of any instrument that would prevent them from slipping into poverty.

These effects are not of secondary order – i.e. determined by the different degree of trust toward single countries financial stability – but rather of primary order as they are driven by the financial markets strong belief that peripheral countries are deprived of any instrument to compensate asymmetries while core ones gain tools supporting internal growth. (Canale et al. 2021 and for similarities with second generation models, Flood and Marion 2000).

The paper is organised as follows: Section 2 focuses on T2 imbalances and the corresponding differences in long-term interest rates (LTR) on government bonds calculated by the ECB for convergence purposes, deserving attention to the direction of causality from T2 to LTR. Section 3 presents the stylised theoretical model to show how the presence of T2 imbalances affects poverty. Section 4 features the empirical investigation. The data are described and the supposed relation is introduced. Section 4.1 presents the methodology and results. Finally, Section 5 presents the conclusions and provides some policy reflections.

## The issue of TARGET2 balances and the effects on interest rates

The Eurozone is comparable to a fixed exchange rate regime. However, because there is only one currency, a particular settlements mechanism, namely TARGET^1^—which evolved into T2 in November 2007—was devised as an alternative to the purchase and sale of foreign exchange reserves. With T2, countries with a balance of payments surplus receive, via their national central bank, the net credit coming from the balance of payments deficit of another country. Deficit countries, in turn, have a net debt with surplus countries, the cost of which is determined by the refinancing rate set by the ECB for the European banking system.

Thus the ECB acts as a supranational monetary institution and uses the T2 mechanism as a multilateral clearing system; each international transaction within the Eurozone leads to a shift in the net creditor/debtor positions in euros of the national central bank involved with regard to the ECB. Therefore, T2 balances lie at the heart of the perfect functioning of the monetary union as, like other payments systems, they allow international payments in any kind of macroeconomic condition and even in the presence of large asymmetric shocks (Barredo-Zuriarrain and Cerezal-Callizo 2019; Lavoie 2015). In this respect, the compensation mechanism acts as a kind of credit line provided to countries experiencing a balance of payments crisis that makes the Euro-area more resilient as a currency union than either the gold standard or more traditional dollar pegs (Klein 2017).

The issue of target imbalances has been at the centre stage of the economic debate since the financial crisis. Some view the T2 system as a way to alter the “rules of the game” or the adjustment mechanisms among countries occurring in a fixed exchange rate regime through the increase in interest rates (Sinn and Wollmershäuser 2012). The T2 system is considered by others to be a way to record discrepancies of an accounting nature, thereby respecting the banking principle operating in a closed economy (Gros 2017; ECB 2016). However, despite the banking nature of external deficits or surpluses, the system fuels the distance between core and periphery through its effect on interest rates. Therefore, the “rules of the game” do not disappear, but rather are destined to be long-lasting, fed by a self-fulfilling mechanism of more (less) resources and lower (higher) interest rates.

Before the 2007 financial crisis, this settlement mechanism between countries functioned well in an integrated capital market. The difference between saving and investment was actually considered a good opportunity for capital from surplus countries to flow towards deficit states in order to gain better returns. (Blanchard and Giavazzi 2002). Public bonds were considered to be safe and the spreads in LTR were almost negligible.

After the crisis, single Eurozone countries were not able to preserve their external equilibrium and T2 balances started to register discrepancies among internal performances. In the first period lasting from 2008 to 2013, once the crisis hit aggregate demand and differences between countries emerged, it was the current account, which became the proxy for financial markets to evaluate a country’s ability to repay its debts (Auer 2014). Countries with a current account deficit experienced outflows of capital and increases in interest rates. National borders became important once again and suddenly T2 had to register discrepancies between components in the balance of payments. Countries like Germany and the Netherlands with current account surpluses experienced massive inflows of capital, while countries like Italy and Spain with current account deficits signalled their inability to pay their debt in the future and negative target balances increased sharply. As a result, long-term yields increased in peripheral countries, while decreasing in their core counterparts (Canale and Marani 2015).

Starting from July 2012, the situation changed because of the policy measures implemented at both European and national level. First, the European Stability Mechanism (ESM) was created. Unlike previous bail-out funds, the ESM is a permanent mechanism with an unlimited lending capacity, allowing peripheral countries to receive financial assistance under the strict condition of implementing Stability and Convergence Programmes. Secondly, many countries introduced the so-called “fiscal compact” in their constitutional law, thereby reducing the room for discretionary fiscal policy measures. Thirdly, the ECB reduced the interest rate on the main refinancing operations until the level of 0.00 points was reached (the marginal lending facility was reduced to 0.25 points and the deposit facility to -0.40 points). Finally, a massive injection of liquidity was implemented through so-called quantitative easing (QE). In particular, the public sector purchase programme (PSPP) allowed government bond yields to decrease and to reduce the pressure on peripheral countries' public accounts. Summing up, fiscal restrictions reduced the current account deficit in peripheral countries via decreased imports, thereby reducing the need to have capital inflows to reach the balance of payments equilibria. At the same time, the easy monetary policy lowered interest rates and absorbed some of the bonds which the market was unwilling to buy.

At first sight, these combined measures—implemented following the announcement of the ECB governor, Mario Draghi, to save the euro “whatever it takes”—reduced differences among countries and were supposed to lay the foundations for a new path towards convergence. From September 2012 to the beginning of 2015 target imbalances declined, signalling a sustainable path towards the balance of payments equilibrium. However, since the beginning of 2015, T2 balances have started to diverge once again and a question has arisen about the deep-rooted origins of these new imbalances. The official interpretation appears to lean towards the explanation that the recent increase in target imbalances is the automatic result of QE. They are of a technical nature since QE is implemented through both the ECB and National Central Banks (NCBs) (Gros 2017; ECB 2016), which are obliged to record in their financial statements any massive purchase of public bonds of countries in difficulty, such as Italy and Spain. Contrastingly, it is also held that, when observing T2 balances from the balance of payments perspective, they reflect the interaction between ECB policy choices and the private sector’s wishes (Dosi et al. 2018). The presence of a T2 imbalance documents imperfect substitutability between internal and external assets and hence is the signal of non-perfectly integrated capital markets (Klein 2017, Minnena 2017).

**Fig. 1:**
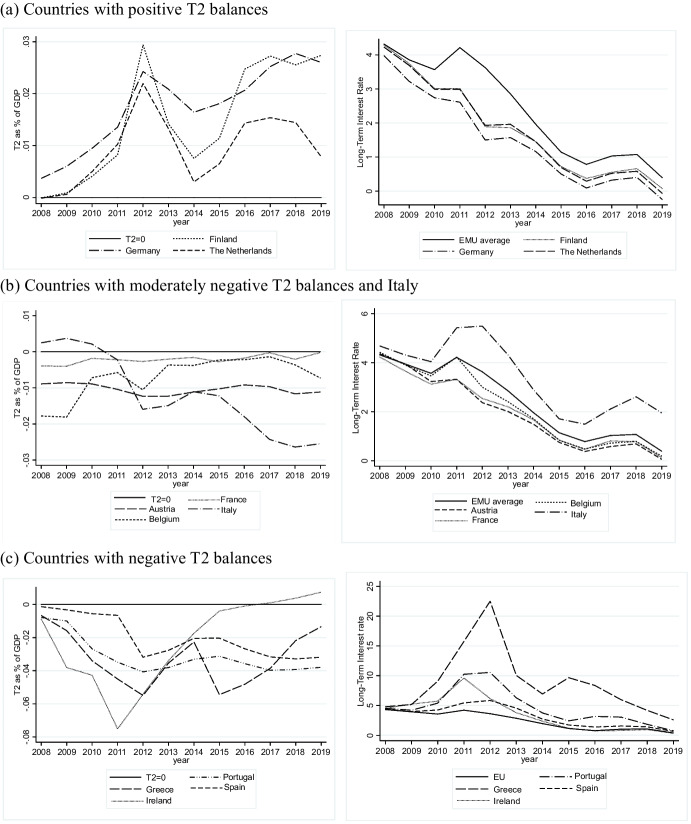
T2 imbalances as a % of GDP and long-term interest rates for convergence purpose in 11 Eurozone countries (2008–2019). Panel (**a**). Countries with positive T2 balances. Panel (**b**). Countries with moderately negative T2 balances and Italy. Panel (**c**). Countries with negative T2 balances. Source: own calculation on ECB and Eurostat data

Detailed observation of the T2 calculated as a percentage of GDP and LTR dynamics in EMU countries from 2008 to 2019 sheds light on the flaws lying behind currency union under a balance of payment crisis. The choice to compare T2 with the LTR depends on the fact that the public debt represents the financial asset belonging exclusively to a state and therefore their spread capture the imperfect substitutability between domestic and foreign securities, whatever the interest rate set by the ECB and the RO implemented. The LTR is the rate that reveals the speculative investors’ behaviour since it is the return of sole asset that can be considered as “national”. In Fig. 1 T2 balances as % of GDP (left) and LTR dynamics (right) are represented for the 11 Eurozone countries considered.

In panel (a), the T2 balances are represented as percentages of GDP and LTR for the so-called core countries. For Germany, the Netherlands and Finland the balance of payments always shows a surplus as the three lines describing the behaviour of T2 are always above T2 = 0. From 2008 to 2012 they show an increasing trend. In the aftermath of the crisis their current account surplus was the signal that their economies were safe and able to ensure safe returns. Capital flowed into the above countries, increasing the amount of resources available within state borders. The common policy measures adopted after 2012 caused a fall in the positive imbalances: the sharp reduction in ECB interest rates, QE and especially the austerity measures implemented in peripheral countries reduced the external imbalances in the strong belief that these combined measures would be enough to preserve the stability of the Euro. However, since 2014 a new increase in positive T2 as a % of GDP has been registered, signalling that something is lacking in the common policy strategy. To the right, the respective LTRs are reported: all three are below the average EMU value, signalling for Germany, the Netherlands and Finland a lower cost for resources to be invested and spent on consumption for the whole period considered, whatever the common policy strategy.

In panel (b) countries with an intermediate position are represented together with Italy. Austria, Belgium and France have a negative but small T2. If we exclude the first two years for Belgium, they all hover around or below -0.01% of GDP with long-term interest rates below and very near the average of the whole currency union. For these three countries therefore very small target imbalances are associated with LTR following more or less the average of the currency union. In particular, France is a big country, with very small target imbalances, while Austria and Belgium are small countries, whose T2 in absolute value is small in respect to other countries. Furthermore, it is worth to be noted that the three countries experience a trajectory toward stability of T2 imbalances. The case of Italy has to be examined separately. In the first two years, positive and moderate T2 are observable together with long-term interest rates very close to, albeit higher than, the average value. From 2010 to 2012 T2 turned from positive into negative and interest rates recorded a sharp increase, high above the EMU average. In subsequent years, Italy registered a reduction in external deficit as a consequence of the austerity measures implemented. They reduced the current account deficit and signalled to investors that the debt would be repaid in the future. Interest rates also decreased, albeit always remaining above average. Since 2015 a new inversion has been detectable on the left-hand-side of panel (b) by increasing negative values of T2, and on the right-hand side by increasing interest rates. It should be recalled that since 2013 the monetary policy has not changed its overall strategy.

Finally, in panel (c) countries with always negative T2 are reported, namely Greece, Ireland, Portugal and Spain. On the left, it is shown that the settlement mechanism registered negative and decreasing values until 2012. The negative values shifted towards zero in the subsequent years. The direction of the movement is persistent for Ireland, which benefited from a financial assistance programme in 2011, after which the country experienced a period of ever-increasing growth able to offset its external imbalances.

For the remaining three countries, another story can be told: for Portugal, the negative external imbalances widened in 2014 and then reduced in 2015, while for Greece and Spain the negative trend is still moving downward. The behaviour of LTRs for the countries considered is consistent with the hypothesis formulated with regard to the relation with T2 imbalances. Until 2012, they increased for all the countries, while in subsequent years they started to decrease and to approach the average of all EMU countries. Predictably, the last values for Ireland are very close to the EMU average.

To sum up, the dynamic of T2 seems to be associated with specific LTR values. In countries with positive T2 the average LTR is always below the average, while in countries with negative T2 imbalances, interest rates stay above the average.^2^

In our interpretation, extending the models about the currency crisis stating that it is private expectations about the future course of the economy that triggers the change in interest rates; it is T2 imbalances that fuelled the increase in long-term interest rates. The internal interest rate is determined by the interaction with the balance of payment equilibrium (Makin and Narayan 2011).

This view is supported by an assessment implemented on the two variables of interest through the PMG estimator (see below for an in depth description of the empirical methodology). The estimation has been implemented in both direction – is T2 to affect LTR or the other way-round? – to evaluate the causality connection. In the econometric design, we included a proxy of the monetary policy stance – the interest rates on safe government bonds—to account for the ECB strategy during the whole period considered. Results are presented in Table 3 in appendix. The coefficient of the variables are all significant and with the expected sign. However, according to the empirical estimates, it is T2 that triggers LTR as the error correction term, shows a significant, negative and lower than one value. It represents the evidence that the two variables are connected in the long run and that the causality connection goes from T2 to LTR. When observing the right section of Table 3, it is evident that, despite the significance of the estimated coefficients, the ϕ_i_ term is not significant, supporting, therefore, the conclusion that the causality relationship cannot be accepted. However, this conclusion does not exclude that the causality relationship can work also the other way round and that there are many other variables feeding target imbalances – among which the current account covers a special role (Auer 2014) – but they seem to be, following our point of view, a consequence of the perceived unsustainability of the Eurozone policy rules.

The estimates also reveals that the effects of T2 on LTR are not eliminated by the easy centralized monetary policy. On the contrary, the T2 imbalances and interest rates divergences remained unaltered or even increased.

## The connection between poverty and target imbalances

The general framework for our stylized representation refers to the second and third generation models about currency (Kydland and Prescott 1977; Obstfeld 1986a and 1986b). These models rely on the idea that the fragility of an exchange rate regime depends on private expectations that pull, ex-post the country into a currency crisis. They anticipate gains and losses associated to the changes in interest rates set by the central bank in the attempt to preserve the parity. They have output effects that might be too costly for the internal equilibrium. Investors' knowledge of these effects feeds speculative behaviours and accelerates through the further reaction of the central bank on interest rates the abandonment of the fixed exchange rate.

Transforming this reasoning so as to apply it to the Euro area, where monetary policy is centralized, it is possible to affirm that T2 imbalances arise as a result of different expected returns, but then, when a balance of payment crisis occurs, amplify, generating a self-fulfilling process of divergence in interest rates and therefore in growth and poverty. Single countries belonging to the monetary union, despite not having a central bank, face a hidden balance of payment crisis (Cesaratto 2017).

To explain how the model works, let us start from a very simple equation defining the equilibrium income on the demand side:

1 $$y=\mathrm\rho\left[A-b(i-\pi^e)-\xi(\pi_I-\pi_E)\right]$$

Equation (1) states that the rate of growth y increases, according to the value of multiplier ρ, as autonomous demand increases, as nominal interest rates *i* decrease or if inflation expectations π^e^ increase. The last term of the equation measures the reaction of the aggregate equilibrium income to the degree of competitiveness measured as the difference between internal π_I_ and external prices π_E_. An equation explicitly defining how internal prices are set is omitted, as it would add little to the working of the model. The nominal exchange rate is not included as the equation refers to a country belonging to a monetary union and its increase or decrease is supposed, for the sake of simplicity, to have homogeneous effects on all the countries belonging to it.

It may be assumed that T2 imbalances initially arise as a result of the difference between internal and external expected rates of growth, for example generated by the planned fiscal retrenchments necessary to comply with fiscal rules during declining macroeconomic conditions (Fatás and Summers 2018). Once capital starts to flow out towards richer countries, the speculative investors’ behaviour affects relative interest rates such that the following holds:

2 $$i={i}_{ECB}-\chi T2$$

According to Eq. 2 the interest rate in each single country is equal to the interest rate the ECB sets, minus a term which is proportional to T2. In other words, in the absence of a national monetary policy designed to preserve the exchange rate, net capital flows produce the same effect on internal interest rates. Even in presence of an high institutional commitment to preserve the parity investors’ behaviour know the absence of nation instruments to preserve output to fluctuate and insist on investing where output is not expected to fall and vice versa (Canale et al. 2021). If T2 balances are positive, the internal interest rate is lower than the EMU average; if T2 balances are negative, the internal interest rate is higher than the EMU average. The hypothesis presented in Eq. (2) is supported by the empirical estimates presented in the previous paragraph and contained in appendix. They are not therefore of secondary order, but driven by the investor’s awareness that national governments cannot use autonomous policy instruments (Canale et al. 2018). The connection presented in Eq. (2) is supposed to be the prevailing phenomenon and does not exclude that divergent interest rates trigger divergent target imbalances: once the mechanism of interplay has started, it is difficult to state which variable come first. However, without shared policy tools, it feeds a self-fulfilling process of divergence.

Absolute poverty is assumed to depend negatively on growth and is described by the following:

3 $$MD={\lambda }_{0}-{\lambda }_{1}y$$

where MD is material deprivation or the measure of absolute poverty, λ_0_ is the component not depending on growth, but rather on national policies, and λ_1_ measures the growth effect on poverty conditions.

Substituting Eq. (2) and (3) in Eq. (1) we have the following relation between absolute poverty and T2:

4 $$MD={\lambda }_{0}-\lambda \rho \left[A-b\left({i}_{ECB}-\chi T2\right)-\xi \left({\pi }_{I}-{\pi }_{E}\right)\right]$$

Hence:

5 $$\frac{\Delta MD}{\Delta T2}=-\lambda \rho b\chi$$

Material deprivation is inversely correlated with the target balance.

This result can be explained through the effect of T2 imbalances on LTR that in turn affect all the other market interest rates inside each single country. Furthermore, inside the policy framework of the Eurozone, interest rates condition national governments ability to use fiscal policy to support internal equilibrium as they affect both additional debt to be issued and the structural public balance adjustment to be implemented to comply with fiscal rules (Fatás and Summers 2018, Blanchard and Leigh 2013). This mechanism fuel divergences inside the Eurozone and deprives (or improves) the national policy tools to be used to reduce poverty (Canale and Liotti 2021; Canale et al. 2019).

## An empirical investigation

The empirical model is built on the hypotheses formulated that target balances are inversely correlated with poverty. The sample contains eleven Eurozone countries: Austria, Belgium, Finland, France, Germany, Greece, Ireland, Italy, the Netherlands, Spain and Portugal. From the 12 Eurozone countries, which adhered to the common currency from the beginning, Luxemburg was removed due to its special features. The time span goes from 2008 to 2019. The year 2008 coincides with the onset of T2 imbalances after the financial crisis. Before then, the balance of payments was almost in equilibrium as current account surpluses or deficits were compensated by capital flows. The year 2019 is the last available observation before the COVID-19 crisis. We are aware that the number of observations is too small to give a strong support to our interpretation. However this inspection can be considered as a first – despite limited – attempt to detect a mechanism of divergence.

Data regarding T2 balances were obtained from the ECB website at http://sdw.ecb.europa.eu/browse.do?node=9691112 where they are posted on a monthly basis. To make the data comparable with “material deprivation” collected annually, the average value of 12 months per country was calculated. Furthermore, to make cross-country comparisons T2 balances were calculated as a percentage of GDP.

Material deprivation is the number of people materially deprived, expressed as a share of the total population. This indicator refers to physical conditions and is based on the availability of specific physical assets (Crettaz 2015). It is therefore more apt – in respect to a measure of inequality as the indicator ‘monetary poverty’ is—to approximate the drain of resources in presence of a balance of payment crisis. It represents the percentage of the population that cannot afford at least four of the following nine items: 1) to pay their rent, mortgage or utility bills; 2) to keep their home adequately warm; 3) to meet unexpected expenses; 4) to eat meat or protein regularly; 5) to go on holiday; 6) a television set; 7) a washing machine; 8) a car and 9) a telephone (Eurostat 2016). Data concerning material deprivation were collected from Eurostat EU-SILC statistics http://ec.europa.eu/eurostat/web/income-and-living-conditions/data/database. Material deprivation represents a measure of extreme poverty based on a minimum level of essential goods that people should have access to, is comparable across countries, and its management and correction can be considered a step towards the construction of a social and economic integrated monetary union (Fusco et al. 2011; Nolan and Whelan 2010). However, to reinforce the validity of the model, we inserted monetary poverty as a control variable to account both for its effect on material deprivation and its relationship with T2 imbalances.

Initial examination of the behaviour of the two indicators can be useful to obtain a straightforward picture of the relation between T2 imbalances and absolute poverty.

**Fig. 2:**
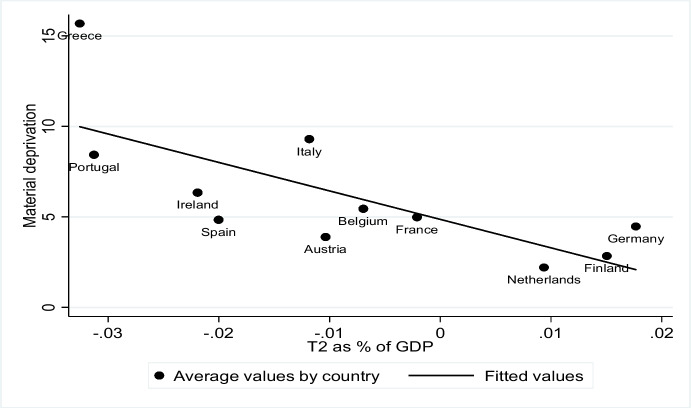
T2 as % of GDP and material deprivation (2008–2019). Source: own elaboration on ECB and Eurostat data

In Fig. 2 for each country, average T2 balances for the whole period considered are compared with average material deprivation. Three main groups of countries can be distinguished: the first including Germany, Finland and the Netherlands is at the bottom right of the figure. They all have positive T2 balances as a percentage of GDP and a corresponding lower level of material deprivation. In the centre of the graph Belgium, France and Austria are depicted: these three countries have moderate negative T2 imbalances and a higher percentage of materially deprived people than the previous group. Italy and Spain also appear in the centre of the graph: Italy as shown in Fig. 1 has a higher balance of payments deficit and long-term interest rates than the EMU average. This led to a greater effect on growth and therefore more people living with real GDP per capita below the absolute poverty threshold. The restrictive fiscal measures implemented to comply with fiscal rules increased such effects. With regard to Spain the relation between material deprivation and T2 imbalances seems to be less pronounced. During the time range considered, in 2012, Spain received financial assistance from the EFSM to save the banking sector. These helped reduce average interest rates to mitigate the effect of reduced growth and negative target imbalances on poverty. The last group of countries appearing on the upper left side of the picture consists of Greece, Portugal and Ireland. Portugal is close to the fitted values: despite receiving financial assistance, the problems of public accounts sustainability induced governments to limit the provision of public services, which in turn had negative effects on aggregate demand and growth. Ireland is a similar case to Spain: initial massive capital outflows occurring soon after the crisis, due to the crisis of the banking sector, were stemmed through financial assistance received in the period 2011–2013. The increase in interest rates was lower and hence the effect on growth was as well. Of all the countries examined, Greece was that with the largest percentage of people living in absolute poverty and the highest negative T2 imbalances. The country received funds almost throughout the whole period considered (from 2010 to 2018) from Eurozone countries, the IMF and the ECB in order to reduce problems related to the sustainability of public sector accounts and the unavailability of private investors to finance the additional public debt. The condition for receiving financial assistance was the implementation of structural public balance adjustment programmes, producing pronounced negative effects on GDP growth (Blanchard and Leigh 2013; Fatás and Summers 2018).

To deepen the analysis other variables that affect absolute poverty (Nolan and Whelan 2007; Hick 2012; Crettaz and Suter 2013) as well as some capturing the policy structure of the Eurozone are inserted into the model one by one. This strategy is necessary to make the empirical model work, as the reduced number of observations limits the possibility to individuate convergence and the existence of a long-run connection. However, when implementing post estimation tests, after linear regression including contemporaneously all the explanatory factors, we found that the null hypotheses of no omitted variables (Ramsey 1969) and no specification error (Pregibon 1980) cannot be rejected. These results does not removes completely the limits of the empirical strategy, but at least allows reflections about the results obtained. The variables are as follows: 1) the interest rates on government bonds rated AAA + extracted for each year from the yield curve with one-year maturity (YC). The choice of this variable is based on its ability to capture the monetary policy strategy as a whole, as it includes both the ECB interest rate setting policy and the effects of open market operations; the yield curve is a key determinant of the financing conditions of the economy and a central element in the transmission of monetary policy. It is affected contemporaneously by policy interest rates and public and private bond purchase programmes and therefore provides information about the true safe rates present in the market and their potential influence on the real economy (Lane 2019; Liotti and Canale 2021). It is supposed to be positively correlated with poverty; 2) per capita GDP (PC_GDP). The higher the per capita income, the lower should be the material deprivation rate (Kis et al. 2015). 3) Monetary poverty (MP), that is the share of population at risk of poverty after social transfers, or living below the threshold of 60% median income. It captures the distance between the middle and the bottom incomes and is therefore supposed to be highly positively correlated with the number of people living in absolute poverty (Kis et al. 2015); 4) the amount of private credit, always as a share of GDP (CR), as an indicator of the presence of financially constrained households and banking system efficiency. The literature affirms that access to credit increases an individual’s opportunity to anticipate future income via, for example, the implementation of an entrepreneurial activity today. However, the excess of private debt, combined with stagnating or falling real wages, increases inequality and poverty (Stockhammer 2015). 5) Structural public balance adjustment (SA). It is the change in structural public balance and is indicative of discretionary policy adjustments (the OECD definition available at https://stats.oecd.org/glossary/detail.asp?ID=3343). When positive, it registers restrictive fiscal policies and, when negative, expansionary ones. Its various flaws derive from its calculations in term of output potential, but the IMF and the EC use it for budgetary surveillance; it is the government public balance component the European institution suggests to reduce or allow to increase to reach the objective of sound public finance. Austerity measures are supposed to exert a negative impact on poverty (Canale et al. 2019). 6) Unemployment (UN). This control variable is introduced following the hypothesis that the higher unemployment is, the higher the number of people living in poverty. 7) The number of people with an upper secondary, post-secondary non-tertiary and tertiary education (levels 3–8), as a percentage of people aged 15–64 (EDU). Education plays a central role in assuring access to the labour market and in gaining a higher income. While the effect on inequality is controversial (Knight and Sabot 1983), the effect on the number of absolute poor people should have an opposite sign (Boarini and D’Ercole 2006). 8) Expenditure in social protection as share of GDP (SE). This consists of transfers, in cash or in kind, to households and individuals to relieve them of the burden of a defined set of risks or needs. Of course, a higher social protection expenditure is expected to reduce material deprivation (Nelson 2013). The inclusion of all these control variables – despite inserted one by one—allows taking into account also their connection with T2 imbalances.^3^ However it should be recognized that a longer period would allow to consider all the variables contemporaneously and release results to be considered of a more solid validity.

### Methodology and results

The empirical strategy aims at testing the existence of a linear relation between T2 balances and material deprivation. The methodology adopted is a subset of dynamic panel data techniques and assumes the form of the pooled mean group (PMG) estimator. This relies on cointegration and the error correction form (EC), and is considered to be consistent for estimating dynamic heterogeneous panels, as the long-run dynamics is assumed to be equal across groups, while in the short run the process of adjustment may vary across the panel members (Pesaran et al. 1997, 1999; Blackburne and Frank 2007). It detects the possible presence of a stable relationship even in the presence of a reduced number of explanatory variables and different dynamics in each country.

The long-run equation is described by:

6 $$M{D}_{i,}{}_{t}={\alpha }_{i}+{\lambda }_{i}M{D}_{i,t-1}+{\beta }_{i,0}T{2}_{i,t}+{\beta }_{i,1}T{2}_{i,t-1}+{\varepsilon }_{i,}{}_{t}$$

where again MD is the poverty rate indicator and T2 represents target imbalances. The error correction equation describing the short-run speed of adjustment is:

7 $$\Delta M{D}_{i,}{}_{t}={\phi }_{i}(M{D}_{i,}{}_{t-1}-{\vartheta }_{i}-{\vartheta }_{1,i}T{2}_{i,}{}_{t})-{\beta }_{i,1}\Delta T{2}_{i,t}+{\mu }_{i,t}$$

It is easy to verify that ${\vartheta }_{i}$ and ${\vartheta }_{1,i}$ are the long-run coefficients calculated as a weighted average of the coefficient of the independent variables. Parameter ${\vartheta }_{1,}{}_{i}$ for the long run and ${\beta }_{1,}{}_{i}$ for the short run are the parameters to be estimated in the model. Parameter ${\phi }_{i}$ is the error correction speed of adjustment. It has to be significant and $-1<{\phi }_{i}\text{ <0}$ must hold. The value and significance of coefficient ${\phi }_{i}$ is of the utmost importance since it confirms the validity of the proposed empirical model: it shows that, in the long run, the dependent and independent variable converge toward a common path and that their difference in trend is progressively decreasing over time.

Since this is a cointegrating technique variables to be inserted in the model needs to be non-stationary in level, stationary when considered in their difference and cointegrated. To choose the appropriate methodology when testing for stationarity and cointegration analysis, the presence of cross-sectional dependence (CD) should be investigated. The cross sectional dependence may derive from an unobservable common shock – a structural break—and a strict interconnection across panel members. In the presence of such a feature, second-generation panel unit root tests should be applied to the dataset. The Pesaran (2004) CD test, performed on our data after a simple panel regression with fixed effects, reveals that the null hypothesis of cross-sectional independence should be rejected (4.813***).

The cross-sectional dependence in series suggests using the so-called “second generation” test to investigate the presence of a unit root in each series or the cross-sectional augmented Dickey-Fuller (CADF) panel unit root test (Pesaran 2007). If the null hypothesis is rejected, the series are stationary. The first rows in Table 1 presents the unit root test results: for the variables in their level the null hypothesis of no stationarity cannot be rejected, while it is rejected when considering the variables at first differences. Therefore variables are integrated of order one I(1).

**Table 1:** Unit root and cointegration second generation tests

Unit root tests and cointegration			
Unit root test			
MD	-1.512	ΔMD	-0.417***
T2	-1.385	ΔT2	-2.853***
Westerlund cointegration test			
Variance ratio		2.9001***	

^***^, **, and * reject the null at 1%, 5% and 10% respectively

To verify the presence of a long-run relationship between the variables considered, the Westerlund (2007) “second generation” cointegration test accounting for cross-sectional dependence is performed. The bottom of Table 1 reports the corresponding results. The null hypothesis of no cointegration is rejected. The presence of cointegration gives strong support to the results of the estimation of the dynamic panel model even in presence of an unknown structural break. Table 2 presents the results of the baseline estimation (model I) and those obtained adding the control variables one by one (models from II to IX).

**Table 2:** Material deprivation and target balances in the Eurozone (2008–2019). PMG estimation results

Variables	I	II	III	IV	V	VI	VII	VIII	IX
Long-run									
T2	-4.421*** (1.292)	-17.346*** (2.707)	-2.146*** (0.553)	-2.559*** (0.428)	-2.254** (1.097)	-3.079** (1.236)	-2.829*** (0.780)	-7.246*** (1.124)	-13.336*** (3.044)
YC		1.741*** (0.423)							
PC_GDP			-0.133*** (0.027)						
MP				0.869*** (0.044)					
CR					-0.161* (0.084)				
SA						0.572*** (0.139)			
UN							0.111*** (0.019)		
EDU								-0.209*** (0.041)	
SE									-0.392*** (0.091)
Short-run									
*ϕi*	-0.369*** (0.066)	-0.233*** (0.054)	-0.522*** (0.197)	-0.741*** (0.121)	-0.379*** (0.089)	-0.372*** (0.102)	-0.488*** (0.164)	-0.355*** (0.089)	-0.288*** (0.099
ΔT2	0.872 (1.800)	0.879 (2.925)	2.310 (3.533)	-0.324 (3.378)	2.058 (2.580)	0.650 (1.486)	1.320 (3.214)	1.832 (2.004)	-2.386 (3.564)
ΔYC		0.205 (0.437)							
ΔPC_GDP			0.368 (0.467)						
ΔMP				0.359*** (0.115)					
ΔCR					0.043 (0.166)				
ΔSA						0.122 (0.173)			
ΔUN							0.191 (0.232)		
ΔEDU								0.015 (0.312)	
ΔSE									0.345 (0.235)
Obs	121	121	121	121	121	121	121	121	121

***, **, and * reject the null at 1%, 5% and 10% respectively: Standard errors are presented below the estimated coefficients;

T2 is target imbalances; YC is the government bond return on safe assets extracted from 1-year maturity yield curve. PC_GDP is per capita GDP, MP is monetary poverty or the number of people living below the threshold of 60% median equivalized disposable income; CR is the amount of credit as percentage of GDP; SA is structural adjustment used as a proxy of discretionary fiscal policy; UN is the unemployment rate; EDU is the number of people having a tertiary education level; SE is social expenditure

The first thing to observe is that the speed of adjustment ϕ_i_ or the way the variables reach the long-run equilibrium is negative, greater than -1 and highly significant in all the proposed models. Whatever the model, in the long run T2 negative (positive) balances increase (decrease) absolute poverty. The value of the coefficient of T2 ranges from -2.146*** when considering GDP_PC to -17.346*** when accounting for monetary policy stance (YC), as consequence of the different connection between the control variables, material deprivation and T2 itself. Furthermore, the coefficient of the control variables appears to be in the long run all significant and with the expected sign.

Indeed, in our model a decrease of interest rates on safe assets decrease poverty (1.741***) and reveals that despite the expansive monetary policy the effect of T2 imbalances on material deprivation is very high (-17.346***) which signals that it fuelled capital flows; opposite sign effects are detected in the long run for GDP per capita (-0.133***), credit (-0.161***), the percentage of people with a tertiary education level (-0.209***) and social expenditure benefits (-0.392***). The sign of the effect of the control variables changes when taking the structural adjustment into consideration: in presence of restrictive fiscal policies (SA > 0) material deprivation increases and the coefficient of T2 also, triggering growing asymmetries across the Eurozone. The expected positive coefficient is confirmed in the case of unemployment, being a proxy of growth. Finally, it is important to note the effect of monetary poverty on material deprivation. The more unequal the distribution of income, the higher the material deprivation (0.869***). This result is in keeping with the prevailing literature, which states that poverty is due to the higher concentration of national wealth in the hands of a few people. It also confirms that T2 imbalances may also affect inequality.

If we exclude the coefficient of monetary poverty, nothing can be said about the dynamic of adjustment in the short run, as the coefficient is not significant. This is due to the heterogeneity of panel members non-uniform dynamics in the adjustment path. However, the validity of these results – despite partial and for a limited time span—are preserved by the fact that variables are cointegrated, it always holds that -1 < ϕ_i_ < 0, and the coefficient of the main variable of interest is always negative. The different magnitude of the coefficients can be explained through the indirect effects each single variable exerts on T2: for example when including YC, the coefficient of T2 is high because of the effect of the monetary policy stance on external imbalances. The same can be said for SE as it meaures the dimension of government interventions to counteract output fluctuations.

The results seem to suggest that, even including control variables, external imbalances have the autonomous capacity to trigger differences in the rate of absolute poverty. This supports the view that, in the absence of centralised compensative instruments, the Eurozone is like a currency area with an irrevocable fixed exchange rate regime (Cesaratto 2017). This autonomous capacity is still valid when the monetary policy stance, the amount of credit that should be highly influenced by the accommodative attitude of the ECB, and the fiscal measures implemented to comply with rules are considered in the estimates. Despite the unlimited availability of reserves, balance of payment crises occur because of the effects capital flows have on internal interest rates, and over which—because of the policy structure of the Eurozone—the ECB has limited control (De Grauwe 2013). However, it should be recognized that this empirical investigation does not allow deriving unequivocal conclusion and that a connection going from interest rates, growth and poverty toward T2 imbalances also provides useful explanation of the existing divergences inside the Eurozone (Auer 2014).

## Conclusions and policy reflections

In the EMU, T2 constitutes a device to make an irrevocable fixed exchange rate regime work. It measures the external deficit or surplus of countries belonging to the currency union. Unlike a mechanism relying on foreign exchange reserves, it has no limits and can potentially sustain the existence of the Euro despite differences amongst members. However, when such differences do exist, it records how they are perceived by investors, whose speculative behaviours entangle countries in a self-fulfilling process of divergence. This paper has shown the effects of this process on the values of the “material deprivation” indicator.

T2 imbalances after the 2007 financial crisis rose to alarming levels. These were the result of current account imbalances, and they predicted divergent rates of growth. When after 2012, monetary policy led to lower interest rates and massive liquidity injections, such differences did not disappear, but it was clear that convergence inside the Eurozone could not be solved through monetary policy alone.

The Eurozone policy framework relies on two main features: a single monetary policy and a fiscal policy based on strict budgetary discipline that is left to be managed by individual states. This model showed its limits in dealing with situations such as the 2007 financial crisis. The crisis also highlighted the greater fragility of some countries relative to others and proved that the economic policy structure was unbalanced.

Since the 2007 crisis, and despite attempts to fix such asymmetries, European policy has not worked. Differences are still evident, and they undermine the currency union. Fiscal retrenchments designed to correct internal and external imbalances have been compromising growth; although they have corrected current account imbalances, they have made the countries concerned to appear less trustworthy in the eyes of the financial markets (Canale et al. 2021). This suggests the need to implement alternative strategies and policy reforms for European institutions.

To ensure convergence and the reduction of poverty, two routes might be followed. The first entails a review of the role of fiscal discipline: budgetary constraints have worn some countries down and left them entangled in a self-fulfilling mechanism of declining growth and increasing fiscal parameters. However, a switch towards a more expansive fiscal stance adopted separately by each country could generate—in the absence of a supportive monetary policy designed to halt speculation—perverse effects on interest rates and ever-increasing debt and deficit.

The second route might involve a mechanism of compensation between negative and positive T2 balances to offset differences between countries. This is not a new idea. It is inspired by Keynes’s proposal (Keynes 1942, in Horsefield 1969) to reform the international monetary system after the Second World War. Part of the responsibility for adjustment would be borne by the creditor country as well as the debtor, and it should be symmetrical. However, or at least this was the case before COVID-19, while retrenchments are imposed on debtor countries to make them respect fiscal parameters, the creditor countries become passive onlookers. Policy reforms should therefore include a set of measures to be adopted by creditor nations, for example fiscal expansion, higher wages, and foreign direct investment in peripheral countries.

The results of the present study could be used to evaluate the possible scenarios and the appropriateness of policy responses to COVID-19. The pandemic originated as a systemic shock but threatens to turn into an asymmetric shock, depending on the degree of countries’ resilience. However, the lessons of the 2007 crisis have not been ignored. The ECB, under Lagarde’s presidency, implemented a set of extraordinary measures—in line with the Draghi’s mandate and with no limits on the accepted collaterals—amongst which the Pandemic Emergency Purchase Programme (PEPP) of €1,350 billion was the most important. A common coordinated fiscal action has been developed in the form of the so-called Recovery Fund, through which European institutions are planning to sustain countries hit by COVID-19. The most innovative aspect of this second instrument is that it is financed through the issue of public debt on behalf of the EC and therefore represents the first true common response to internal tensions. However, the future is uncertain. Fiscal rules have been suspended and monetary policy might switch toward a less accommodative strategy. However, over the past decade as a whole, there has been a growing consensus on the need to implement shared measures to end absolute poverty.

## Data Availability

The data that support the findings of this study are available from the corresponding author, R. R. Canale, upon request.

## Appendix

**Table 3:** Long term interest rates and target balances: two-way PMG estimation results

Long-run			
Dependent variable: LT rates		Dependent variable: TARGET 2	
T2	-80.476*** (8.560)	LTR	-0.002*** (0.000)
YC	2.944*** (0.169)	YC	0.000 (0.001)
Short-run			
ϕ_i_	-0.178** (0.081)	ϕ_i_	-0.111 (0.111)
ΔT2	-57.346** (22.411)	ΔLTR	-0.005*** (0.001)
ΔYC	0.321*** (0.100)	ΔYC	0.002*** (0.000)
Number of groups	11	Number of groups	11
Observations	121	Observations	121

^***^, **, and * reject the null at 1%, 5% and 10% respectively. Standard errors are presented below the estimated coefficients. T2 is target imbalances; LTR is 10 years government bond yields for convergence purpose; YC is the government bond return on safe assets extracted from 1-year maturity yield curve as a proxy of monetary policy stance

## Footnotes

1 Trans-European Automated Real-Time Gross Settlement Express Transfer System.

2 This interpretation complies with the one of those attributing the eruption of the Eurozone crisis after the financial crisis to a sudden reversal of capital flows despite until that moment internal and external assets were considered as perfect substitute (Eichengreen 2010, De Grauwe and Ji 2013, Gros 2012, Alessandrini et al. 2012, Berger and Nitsch 2010).

3 Data on GDP_PC, CR, MP, UN, EDU and SE are available at the Eurostat website (https://ec.europa.eu/eurostat/web/main/data/database), while data on SA are calculated on the basis of the structural balance, retrieved from the IMF website (https://www.imf.org/en/Publications/WEO/weo-database/2021/April).

## Declarations

### Author statement

Rosaria Rita Canale conceived the presented idea, developed the theory, performed the main computations and wrote the manuscript. Giorgio Liotti verified the analytical methods, performed further computations and checked the text.
